# Clinical characteristics of patients with heart failure and intracardiac thrombus

**DOI:** 10.3389/fcvm.2022.934160

**Published:** 2022-10-06

**Authors:** Mei Zhai, Liyan Huang, Lin Liang, Pengchao Tian, Lang Zhao, Xuemei Zhao, Boping Huang, Jiayu Feng, Yan Huang, Qiong Zhou, Yuhui Zhang, Jian Zhang

**Affiliations:** State Key Laboratory of Cardiovascular Disease, Heart Failure Center, National Center for Cardiovascular Diseases, Fuwai Hospital, Chinese Academy of Medical Sciences & Peking Union Medical College (CAMS & PUMC), Beijing, China

**Keywords:** intracardiac thrombus, heart failure, cardiomyopathy, anticoagulants, left ventricular dysfunction

## Abstract

**Background:**

Heart failure (HF) patients are in a hypercoagulable state that predisposes them to an intracardiac thrombus. We aim to assess the clinical features of patients with HF and intracardiac thrombus.

**Methods:**

Patients diagnosed with HF with intracardiac thrombus were enrolled in this study. Patients' demographics, clinical comorbidities, laboratory tests, and cardiac imaging parameters are recorded. Baseline characteristics are described; the relationship between intracardiac thrombus location and cardiac underlying diseases, New York Heart Association (NYHA) class, and left ventricular ejection fraction (LVEF) are analyzed; and the anticoagulation rate is summarized.

**Results:**

A total of 1,248 patients were included in the study. Most patients were men (72.2%) with a mean age of 54 years. The left ventricle is the most frequently involved (66.8%), and the prevalence of left ventricular thrombus is more in patients complicated with coronary artery diseases, ventricular noncompaction cardiomyopathy, and dilated cardiomyopathy (86.3%, 86.4%, and 78.2%, respectively). When combined with atrial fibrillation, atrial flutter, hypertrophic cardiomyopathy, restrictive cardiomyopathy, or valvular cardiomyopathy, the intracardiac thrombus is mostly likely to occur in the left atrium. The incidence rate of left cardiac thrombosis increased with the decline of LVEF, an increase of NYHA class, and enlargement of a cardiac chamber. Overall, the anticoagulation rate was 56.8%, with warfarin still the mainstay drug (45.1%), while the prescription of non-vitamin K antagonist oral anticoagulants rose year by year. As for imaging modalities for thrombus detection and diagnosis, transthoracic echocardiography was the most widely performed (75.1%).

**Conclusion:**

This study summarizes the underlying disease constitution, thrombus location and related factors, imaging modalities, and antithrombotic profile in HF patients with intracardiac thrombus comprehensively.

Intracardiac thrombi are frequently reported in patients with underlying cardiovascular diseases and are commonly discovered at autopsy. In an analysis of 11,724 autopsy cases carried out by Pradeep Vaideeswar et al., 276 (2.4%) had intracardiac thrombus (1). Emerging imaging techniques, especially the extensive application of echocardiography, cardiac computed tomography (CTA), and cardiovascular magnetic resonance (CMR), play an imperative role in the screening, diagnosis, and surveillance of thrombus, and thus largely improved intracardiac thrombus detection and management (2). Heart failure (HF) patients are predisposed to intracardiac thrombosis due to cardiac structure and function abnormality in association with multiple comorbidities. It is reported that thromboembolic events in chronic HF patients range from 1 to 3% per year (3), with intracardiac thrombus being one of the premier causes. Severe systemic arterial embolism events in HF patients and resultant morbidity and mortality caused by intracardiac thrombus necessitate a study featuring clinical characteristics in this population.

By assessing the clinical characteristics and thrombus features of HF patients admitted to our center, this study aims to give insights into the clinical characteristics of patients with HF and intracardiac thrombus.

## Materials and methods

### Study design and patient inclusion

This is a single-center retrospective cohort study. We consecutively enrolled patients hospitalized in the internal medical department of Fuwai Hospital, CAMS & PUMC, from October 2008 to May 2018 who were meticulously examined with inclusion and exclusion criteria. Demographics, clinical characteristics, laboratory profiles, recorded comorbidities, use of antithrombotic agents, and imaging parameters, such as intracardiac thrombus location, cardiac structure, and cardiac function, were retrospectively collected. Intracardiac thrombus location was described in the imaging reports. Cardiac structure and function were assessed by transthoracic echocardiography (TTE).

Inclusion criteria are as follows: (1) diagnosed as HF according to the Chinese Guideline for the Diagnosis and Treatment of Heart Failure 2018 (3), and (2) intracardiac thrombus visualized in echocardiography, CMR, or computed tomography (CT) in any of the chambers including left and right ventricle, left and right atrium, or left atrial appendage.

Exclusion criteria are the following: (1) age under 14 years, and (2) baseline data incomplete.

### Data collection

Baseline data, including age, sex, body mass index (BMI), systolic blood pressure (SBP) at admission, diastolic blood pressure (DBP) at admission, heart rate (HR) at admission, underlying cardiovascular diseases, major comorbidities, New York Heart Association (NYHA) functional class, results of blood routine, liver function, kidney function, and other blood tests, were collected along with thrombus-related indexes, such as size and location reported on echocardiography/CMR/CT, cardiac chamber diameter, and left ventricular ejection fraction (LVEF) reported on echocardiography.

### Statistical analysis

Continuous variables were shown as mean values and standard deviations, or medians and interquartile ranges as appropriate. Categorical variables were presented as frequency and percentage. Continuous variables were compared between the groups with Student's *t-*test. Categorical variables were compared by the Chi-square test. A trend test was applied among the groups.

All statistical tests were two-tailed, and *P* < 0.05 were considered to be statistically significant.

The analysis was performed with the use of SAS software, version 9.4 (SAS Institute, Cary, North Carolina), and R 4.0.3 (Vienna, Austria) statistical software.

## Results

### Baseline characteristics

Baseline characteristics are detailed in Table 1. The study population consisted of 1,248 HF patients with intracardiac thrombus. Most patients were men (72.2%) with a mean age of 54 years. The most common complication was coronary artery disease (CAD) (57.5%), followed by hypertension (39.4%), mainly stage 3 hypertension (53.3%). Non-ischemic cardiac disease accounted for 36.2%, dilated cardiomyopathy (DCM) for 19.1%, valvular heart disease for 16.9%, and atrial fibrillation (AF) or atrial flutter for 35%. More than 50% of the patients underwent guideline-directed management and therapy of HF.

**Table 1:** Baseline characteristics of heart failure patients with intracardiac thrombi.

**Parameter**	
Age (y, mean ± SD)	53.9 ± 14.3
Male [no. (%)]	901 (72.2)
BMI (kg/m^2^, mean ± SD)	24.6 ± 4.0
Heart rate (bpm, mean ± SD)	80.7 ± 18.3
Systolic blood pressure (mmHg, mean ± SD)	115.7 ± 18.4
Diastolic blood pressure (mmHg, mean ± SD)	73.3 ± 12.5
Serum creatinine (μmol/L, mean ± SD)	93.8 ± 33.6
ALT [IU/L, median (interquartile range)]	26 (17,45)
AST [IU/L, median (interquartile range)]	24 (19,34)
Hemoglobin (g/L, mean ± SD)	141.8 ± 20.6
Platelet count (*10^∧^9/L, mean ± SD)	205.7 ± 75.8
Hematocrit (%)	42.6 ± 5.9
Complications	
CAD [no. (%)]	717 (57.5)
Non-ischemic heart disease [no. (%)]	452 (36.2)
Dilated cardiomyopathy ^a^ [no. (%))	239 (19.1)
Valvular cardiomyopathy ^b^ [no. (%)]	211 (16.9)
Atrial fibrillation/atrial flutter [no. (%)]	436 (35.0)
Paroxysmal/sustained ventricular tachycardia [no. (%)]	177 (14.2)
Hypertension [no. (%)]	492 (39.4)
Stage 3 hypertension [no. (%)]	262 (53.3)
Diabetes [no. (%)]	271 (21.7)
Chronic kidney disease [no. (%)]	138 (11.6)
Malignancy [no. (%)]	7 (0.6)
Medication at discharge	
Digoxin [no. (%)]	436 (34.9)
Beta-blockers [no. (%)]	976 (78.2)
RAAS inhibitors ^c^ [no. (%)]	656 (52.6)
Spironolactone [no. (%)]	809 (64.8)
Loop diuretics [no. (%)]	954 (76.4)

Continuous variables are presented as mean ± SD or median (interquartile range). Categorical variables are presented as numbers and frequencies. SD, standard deviation; BMI, body mass index; CAD, coronary artery disease; ALT, alanine aminotransferase; AST, aspartate aminotransferase; RAAS, Renin-Angiotensin-Aldosterone System.

a. Dilated cardiomyopathy: including idiopathic dilated cardiomyopathy, perinatal cardiomyopathy, and alcoholic cardiomyopathy.

b. Valvular cardiomyopathy: including rheumatic valvular heart disease and degenerative valvular heart disease.

c. Including ACEI, ARB, and ARNI.

### Intracardiac thrombus location features and related factors

**Figure 1:**
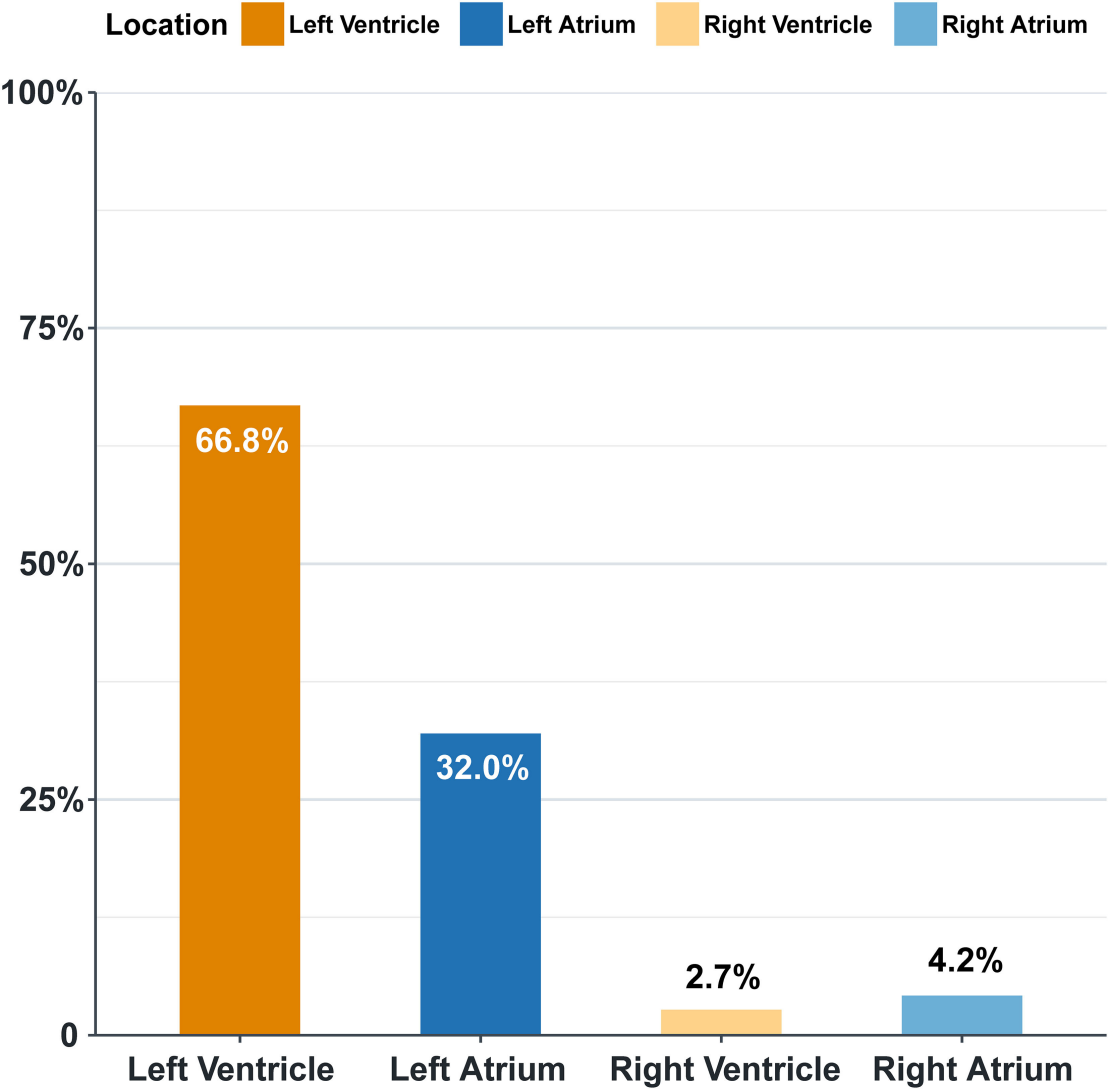
Intracardiac location features in heart failure patients (%).

Overall, the prevalence of left-sided intracardiac thrombi is more than right-sided ones, and the ventricular ones constitute the majority. The left ventricle is the most frequently involved among the four chambers (66.8%), followed by the left atrium (32%) (Figure 1).

The characteristics of HF patients grouped by location are presented in Table 2. In patients with HF and CAD, the intracardiac thrombus is more likely to occur in the left ventricle (86.3%). When combined with anterior myocardial infarction or ventricular aneurysm, the incidence of left ventricular thrombosis is higher (96.2 and 98.2%, respectively). The left ventricular apical thrombus is most likely to be detected in patients with anterior myocardial infarction (77.7%). The left ventricle is predominantly involved in HF patients complicated with ventricular noncompaction cardiomyopathy and DCM (86.4 and 78.2%, respectively). In HF patients combined with AF, atrial flutter, hypertrophic cardiomyopathy (HCM), restrictive cardiomyopathy (RCM), or valvular cardiomyopathy, intracardiac thrombus mostly occurs in the left atrium. The incidence of right atrial thrombus is the highest in HF patients with RCM (32.4%).

**Table 2:** Distribution features of intracardiac thrombi in heart failure patients.

	**Left ventricular thrombi**	**Left atrial thrombi**	**Right ventricular thrombi**	**Right atrial thrombi**
CAD (*n =* 717)	619 (86.3)	99 (13.8)	8 (1.1)	6 (0.8)
Anterior myocardial infarction (*n =* 521)	501 (96.2)	22 (4.2)	3 (0.6)	3 (0.6)
Ventricular aneurysm (*n =* 390)	383 (98.2)	4 (1.0)	2 (0.5)	1 (0.3)
Atrial fibrillation/atrial flutter (*n =* 436)	89 (20.4)	321 (73.6)	3 (0.7)	23 (5.3)
Ventricular noncompaction cardiomyopathy (*n =* 22)	19 (86.4)	2 (9.1)	0 (0)	1 (4.55)
Dilated cardiomyopathy (*n =* 243)	190 (78.2)	37 (15.2)	3 (1.2)	13 (5.3)
Hypertrophic cardiomyopathy (*n =* 30)	8 (26.7)	22 (73.33)	0 (0)	0 (0)
Restrictive cardiomyopathy (*n =* 37)	7 (18.1)	25 (66.6)	0 (0)	12 (32.4)
Valvular cardiomyopathy (*n =* 209)	15 (7.2)	190 (90.9)	0	6 (2.9)
NYHA functional class				
I (*n =* 118)	90 (76.23)	34 (28.8)	0 (0)	1 (0.9)
II (*n =* 346)	189 (54.7)	155 (44.8)	0 (1.2)	7 (1.7)
III (*n =* 311)	177 (56.9)	126 (40.5)	1 (2.3)	18 (4.5)
IV (*n =* 257)	170 (66.2)	76 (29.6)	2 (3.1)	25 (5.5)
LVEF (%)				
≥50 (*n =* 393)	139 (35.4)	241 (61.3)	3 (0.8)	17 (4.3)
35 ≤ LVEF < 50 (*n =* 418)	336 (80.4)	85 (20.3)	0 (0)	11 (2.6)
< 35 (*n =* 413)	346 (83.8)	64 (15.5)	2 (0.5)	21 (5.1)

Abbreviations and annotations as in Table 1.

NYHA, New York Heart Association; LVEF, left ventricular ejection fraction.

**Figure 2:**
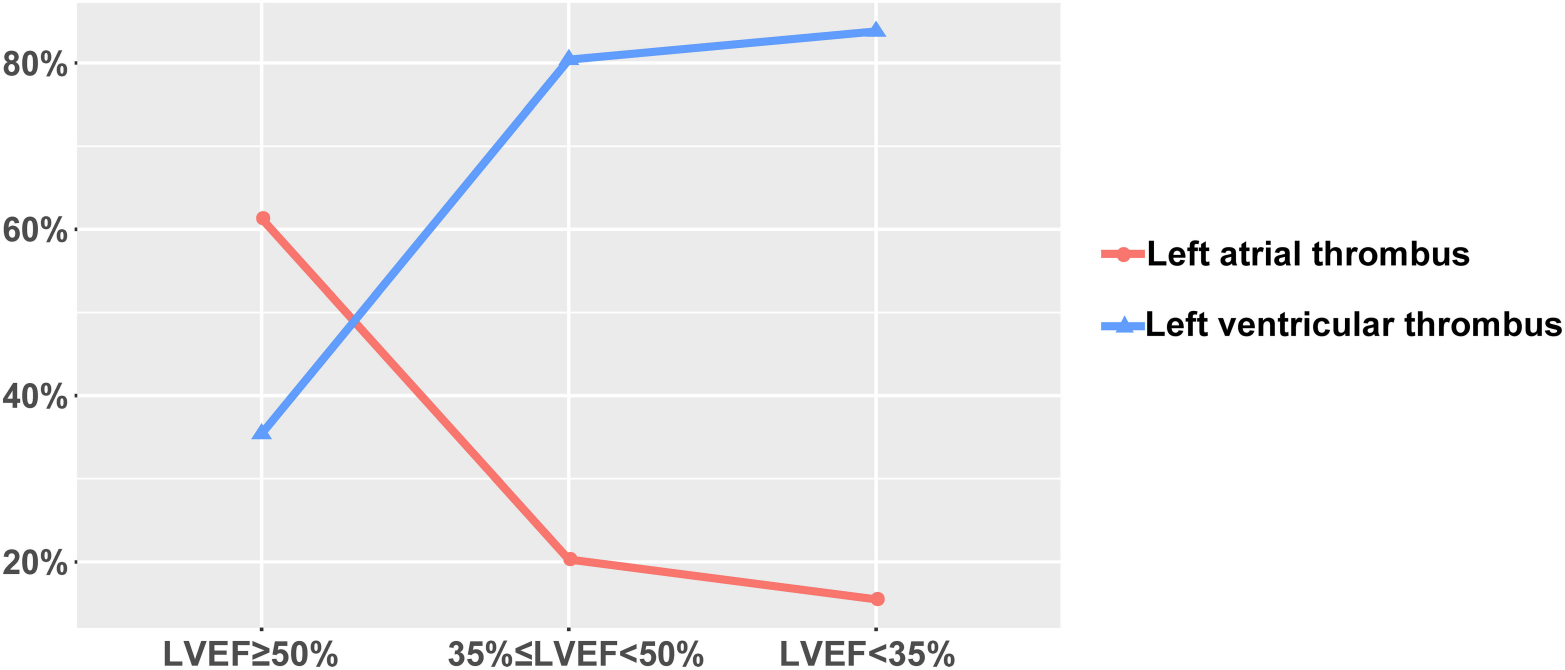
Left ventricular/atrial thrombus incidence rate according to LVEF phenotypes (%).

The proportion of patients with poor cardiac function (NYHA II, III, or IV, or LVEF < 50%) is found to be high in our study population, as is shown in Table 2. The higher the NYHA functional class, the higher the incidence of left ventricular thrombosis and right atrial thrombosis, and the lower the incidence of left atrial thrombosis (*P-value* for trend all < 0.0001). However, no such trend is observed in the right ventricular thrombosis, possibly due to the extremely low incidence rate (*P-value* for trend > 0.05). Analogously, the incidence rate of left ventricular thrombosis increased with the decline of LVEF, whereas the opposite pattern is true for the left atrial thrombus (*P-value* for trend all < 0.0001) (Figure 2). A similar trend was not found in the right-sided thrombus (*P-value* for trend > 0.05).

**Figure 3:**
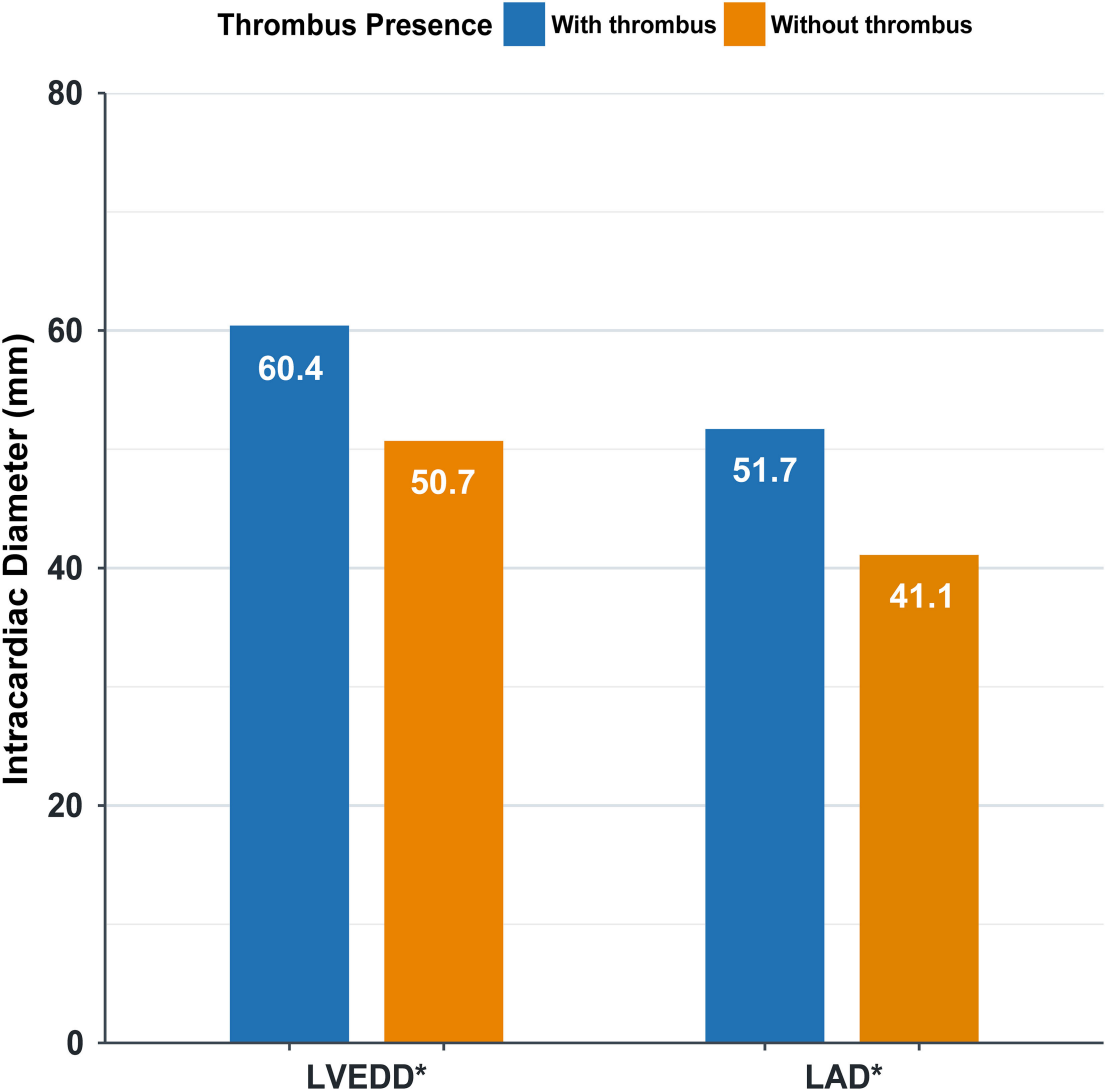
Intracardiac diameter in patients with and without thrombus. LVEDD, left ventricular end-diastolic diameter; LAD, left atrial diameter; * *P* < 0.0001.

Furthermore, left atrial diameter (LAD) is associated with left atrial thrombosis, and left ventricular end-diastolic diameter (LVEDD) is associated with left ventricular thrombosis. Cardiac diameter is significantly higher in patients with intracardiac thrombus (*P* < 0.0001) (Figure 3).

### Antithrombosis in intracardiac thrombus patients

**Figure 4:**
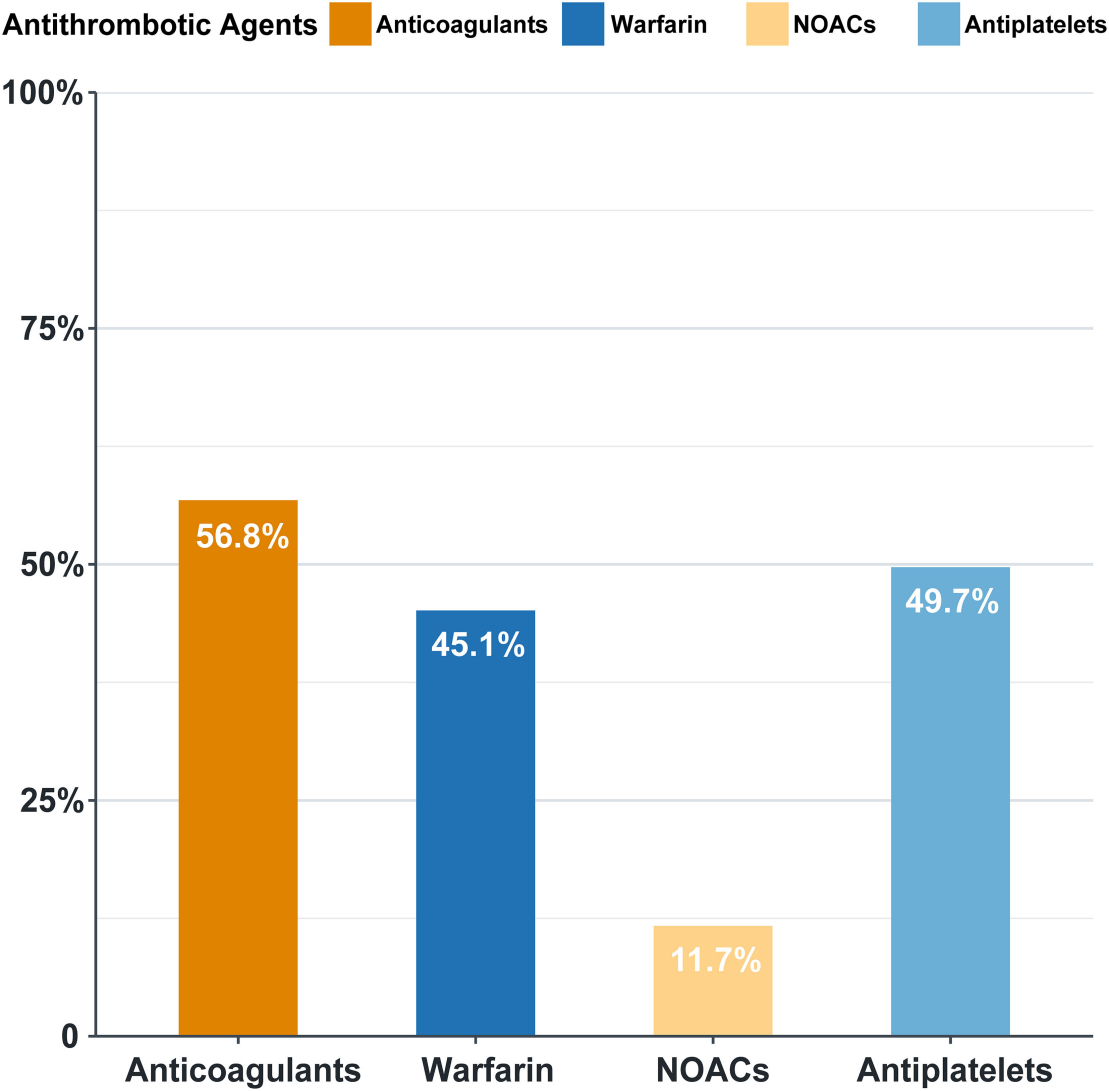
Antithrombotic agent prescription in patients with intraxardiac thrombus (%). NOACs, non-vitamin K antagonist oral anticoagulants, including rivaroxaban, dabigatran, and apaxiban. Antiplatelets: including aspirin and clopidogrel.

**Figure 5:**
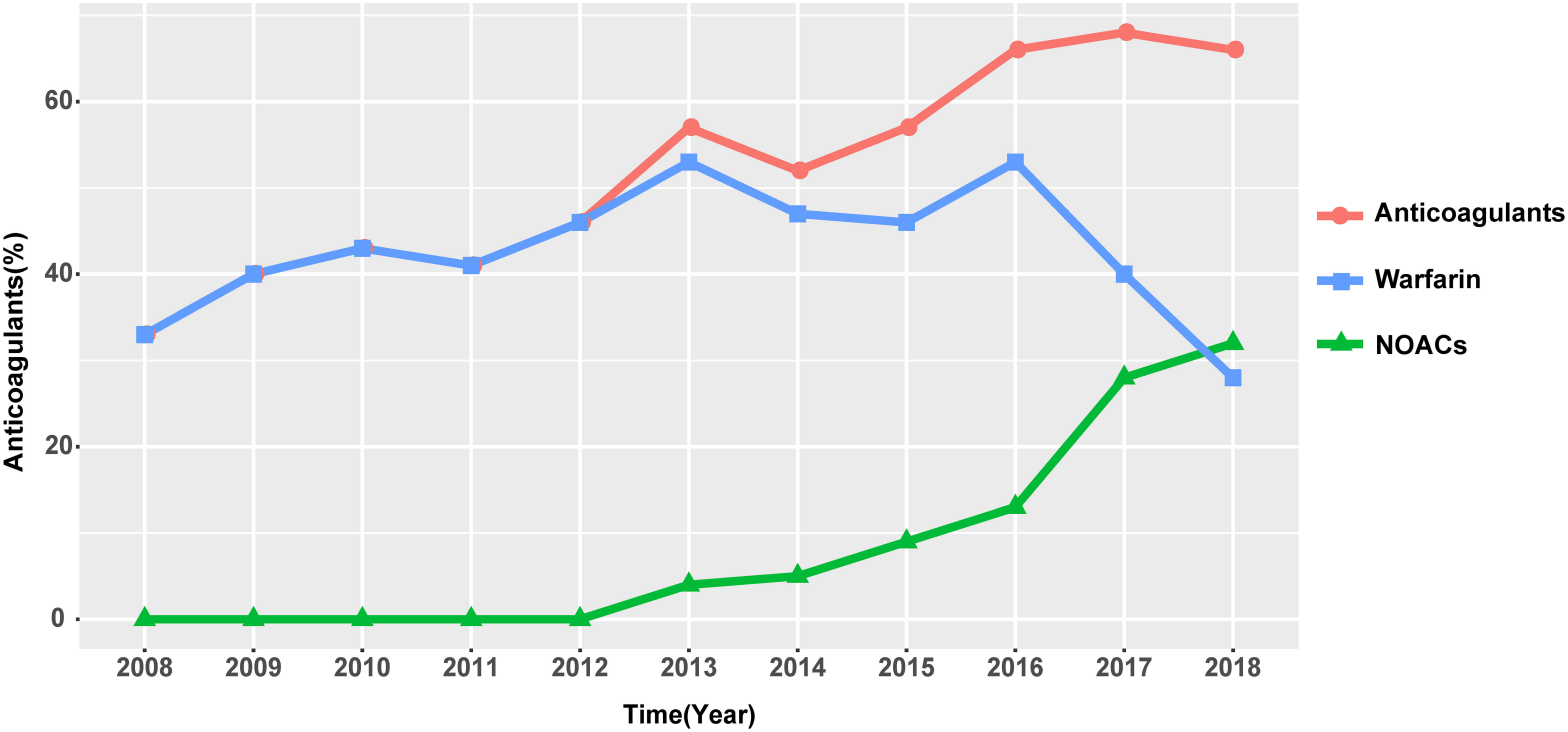
Trend of anticoagulant usage over time. Abbreviations as in Figure 4.

In our study, the overall anticoagulation rate was 56.8%, among which warfarin was the main treatment (45.1%). Antiplatelets were prescribed in 49.7% of the patients (Figure 4). The administration of non-vitamin K antagonist oral anticoagulants (NOACs) and warfarin differs with time (Figure 5). Before 2012, the anticoagulant rate in patients with HF and intracardiac thrombus was < 50%, with warfarin being the only option. However, since 2012, the rate increased year by year with an increasing proportion of NOAC prescriptions and a gradual decline in the warfarin prescription. In 2018, the use of NOACs exceeded that of warfarin.

The comparison of patients on and off oral anticoagulation revealed that patients off oral-anticoagulation were older (55.2 ± 14.0 vs. 52.9 ± 14.5, *P* < 0.01) and were inclined to concomitant antiplatelet use (76.3 vs. 29.5%, *P* < 0.01). There was no significant difference in liver function and renal function between patients on and off oral anticoagulation (Table 3).

**Table 3:** Comparison between heart failure patients complicated with intracardiac thrombus on and off oral anticoagulation.

	**On oral anticoagulation^a^** ** (*n =* 709)**	**Off oral anticoagulation** ** (*n =* 539)**	**P value**
Age (y, mean ± SD)	52.9 ± 14.5	55.2 ± 14.0	< 0.01
Platelet count (*10^∧^9/L, mean ± SD)	203.6 ± 76.4	208.6 ± 75.0	>0.05
Hemoglobin (g/L, mean ± SD)	141.9 ± 21.3	141.53 ± 19.6	>0.05
Alkaline phosphates (IU/L, mean ± SD)	79.9 ± 36.3	79.5 ± 53.9	>0.05
Total bilirubin (μmol/L, mean ± SD)	25.7 ± 18.0	25.2 ± 29.6	>0.05
Direct bilirubin (μmol/L, mean ± SD)	6.8 ± 7.5	7.2 ± 14.3	>0.05
Creatinine clearance rate ^b^ (mL/min*1.73 m^2^, mean ± SD)	75.4 ± 22.7	75.2 ± 24.5	>0.05
Antiplatelets ^c^ [*n*, (%)]	209 (29.5)	411 (76.3)	< 0.01

a. Including warfarin, rivaroxaban, dabigatran, and apixaban.

b. Creatine clearance rate is calculated according to the Cockcroft-Gault equation.

c. Including aspirin and clopidogrel.

### Imaging modalities for thrombus detection and diagnosis

**Figure 6:**
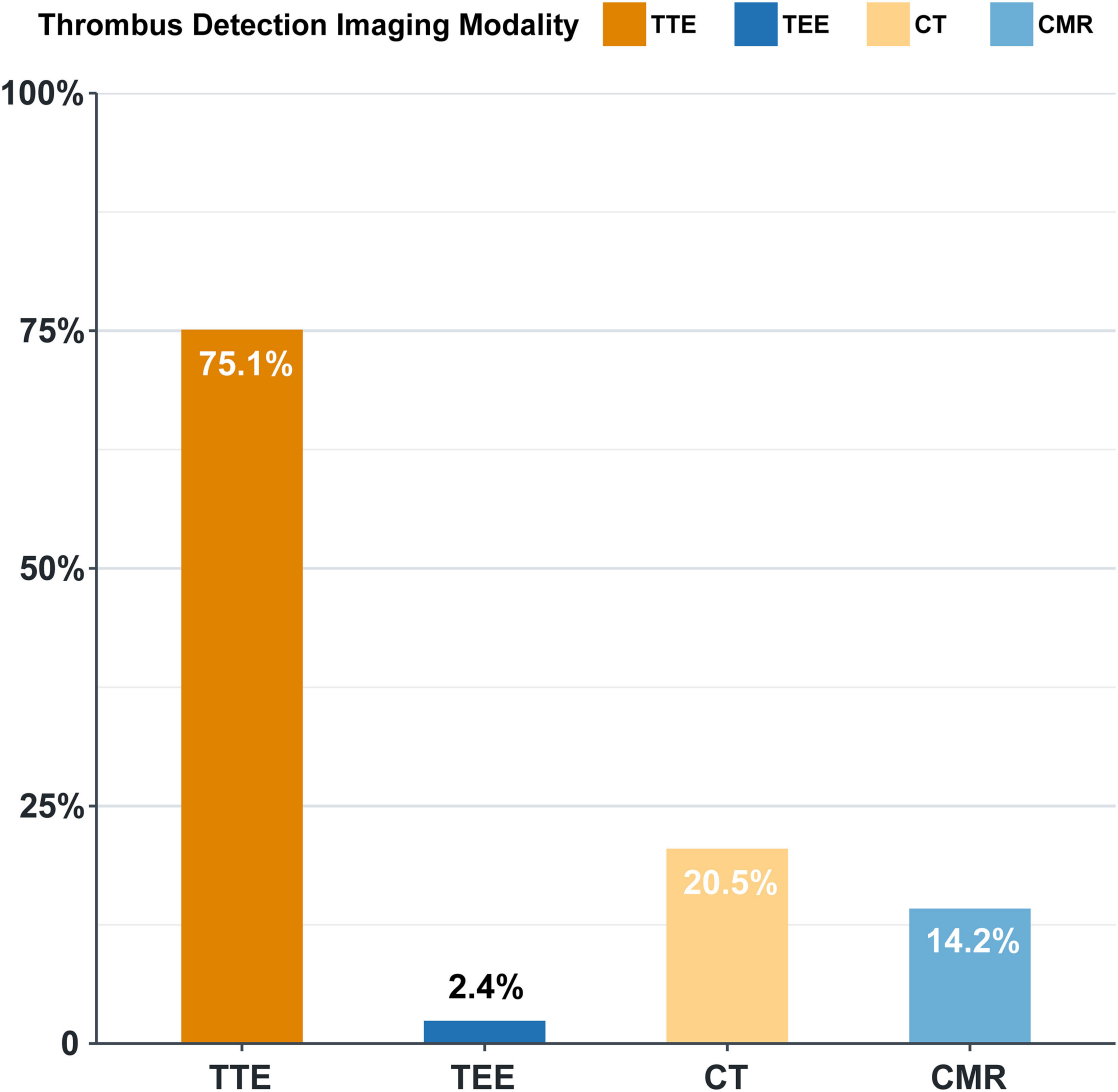
Diagnostic approach of intracardiac thrombus (%). TTE, transthoracic echocardiography; TEE, transesophageal echocardiography; CT, computed tomography; CMR, cardiovascular magnetic resonance.

Transthoracic echocardiography was the most widely performed modality for intracardiac thrombus diagnosis (*n* = 937, 75.1%), followed by CT (*n* = 256, 20.5%), while other imaging modalities accounted for only a small proportion (Figure 6). Transesophageal echocardiography (TEE) was performed in 31 cases, where 30 cases had left atrial appendage thrombus, of which 26 cases were missed by TTE. Of the 209 cases detected by CT and missed in TTE, 163 (78.0%) were diagnosed as left atrial thrombus, 41 (19.6%) were diagnosed as left ventricular thrombus, and 140 (67.0%) were diagnosed as left atrial appendage thrombus.

## Discussion

Heart failure is the end stage of various cardiovascular diseases and has now become one of the most important public health problems in China and around the world. It is estimated that there are more than 37 million HF patients worldwide, causing a major strain on the healthcare systems (4). Intracardiac thrombus is an important complication in patients with HF and is associated with worse outcomes. Pathophysiology mechanisms, such as neurohormonal activation, endothelial dysfunction, proinflammatory cytokine production, increased plasma viscosity, platelet activation, high levels of pro-coagulant factors, and thrombin formation, cause a hypercoagulable state in HF (5), which combined with dilated cardiac chamber and decreased cardiac function predispose HF patients to coagulation activation and fibrin aggregation, subsequently leading to intracardiac thrombosis (6).

### Underlying disease and intracardiac thrombus

In the present study, CAD was the most prevailing underlying disease (57.5%), and non-ischemic heart disease accounted for 36.2%, consistent with the overall etiology constitution in China. Compared to the China Heart Failure Registry Study, in which patients with CAD accounted for 49.4% of the hospitalized patients with HF and non-ischemic heart disease accounted for 23.1% (7), our study includes a combined population with an evidently higher proportion of CAD and non-ischemic heart disease patients, indicating an increased risk of intracardiac thrombosis in these patients.

### Coronary heart disease and intracardiac thrombosis

It is estimated that the incidence of thrombosis following myocardial infarction is 5–10% (3). A previous study reveals that anterior myocardial infarction is accompanied by a significantly higher risk of intracardiac thrombosis, especially in the left ventricle. A study included 100 patients with acute myocardial infarction and LVEF < 40%, and they underwent CMR before discharge (mean scanning time of 4.2 days after admission). It was observed that left ventricular thrombus was found in 15% of the patients, all of whom were diagnosed with acute anterior myocardial infarction (8). Another study of 150 patients with left ventricular systolic dysfunction (LVEF < 40%) drew a conclusion that male gender, prior myocardial infarction, presence of an apical aneurysm, and ischemic scar were associated with thrombosis (9). Consistent with prior studies, our study also reported a remarkably higher risk of intracardiac thrombosis (with the left ventricular apex being the most susceptible) in anterior myocardial infarction patients, especially when complicated with a ventricular aneurysm.

Our study is indicative of future clinical practice. More attention should be paid to patients who are vulnerable to intracardiac thrombus. In particular, key emphasis should be laid on the screening of left ventricular apical thrombus. It also demonstrated the necessity of studies focusing on intracardiac thrombus prevention after myocardial infarction.

### Cardiomyopathy and intracardiac thrombosis

Cardiomyopathy patients are predisposed to HF and intracardiac thrombus.

Increased LVEDD (>60 mm) and low LVEF are independent predictors of intracardiac thrombosis in cardiomyopathy patients (10). In the present study, left ventricular thrombus is the major type of intracardiac thrombus in DCM patients (*n* = 239, 78.2%) (including idiopathic dilated cardiomyopathy, perinatal cardiomyopathy, and alcoholic cardiomyopathy), in line with the previous literature.

Left atrial thrombus is the major type of intracardiac thrombus in HCM and RCM patients. HCM is characterized by left ventricular diastolic dysfunction and wall thickness, attributable to the development of AF. This link is manifested by a high prevalence of AF in HCM, ranging from 17 to 28% (11). Left atrial thrombosis and thromboembolism events associated with AF are more common in HCM when compared to other diseases, and it has been reported that the overall prevalence of thromboembolism in HCM patients with AF was 27.09% (12). In the present study, a considerable proportion (91.3%) of patients with HCM and AF developed left atrial thrombus. HCM is relatively rarely complicated with ventricular thrombus, despite a higher risk of left ventricular thrombosis when complicated with apical aneurysm (12). Similar to the prior studies, all five cases with HCM and ventricular aneurysm in our study developed left ventricular thrombus. The pathophysiological characteristics of RCM are analogous to those of HCM. With ventricular diastolic dysfunction and bilateral atrial enlargement, RCM is prone to atrial thrombosis too (13, 14). In the present study, RCM patients are mainly complicated with bilateral atrial thrombus. What is more, it is worth noticing that right atrial thrombus, hardly seen in other cardiomyopathies, is more prevalent in RCM.

### Cardiac function and structure in patients with intracardiac thrombus

In a study carried out by Mccarthy et al., HF was the most common precipitating factor of intracardiac thrombosis (68.5%), and 86% of the patients were complicated with reduced EF (LVEF ≤ 40%) (15). Similarly, in our study, we observed a close association between thrombus location and cardiac function, and patients with high NYHA functional class are more likely to develop thrombus in the left ventricle and are less likely to develop thrombus in the left atrium. The mechanisms are unclear, but we inferred that intracardiac hemodynamics disorder brought about by cardiac dysfunction and low LVEF plays a pronounced part in the hypercoagulable state within the ventricle.

In our study, there is an association between cardiac chamber diameter and thrombus incidence. Compared to the thrombus-free population, those with thrombus are more prone to have a dilated cardiac chamber. Rarely reported, the association between cardiac diameter and intracardiac thrombosis risk is far from being completely unveiled. A study of 45 patients with DCM of ischemic or idiopathic etiology with mild to moderate systolic dysfunction in sinus rhythm demonstrated that LVEDD was correlated with left ventricular thrombosis (r = 0.38, *P* = 0.005) (16). It is speculated that cardiac chamber enlargement and resultant turbulence formation are conducive to thrombosis.

### Imaging modalities in the detection of intracardiac thrombus

The present study shows that TTE is the most important method in thrombus detection in HF patients, which is also the most commonly used method for the evaluation of cardiac structure and function. Compared to TEE, CT, and CMR, TTE is economically friendly, radiation-free, non-invasive, and widely accepted as the primary screening test for thrombus detection. TEE is more accurate and is the first choice in left atrial appendage thrombus detection. However, for operators, TEE requires higher operating skills; for some patients, it increases the pain to a certain extent. The above-mentioned factors may limit the wide application of TEE in the clinical setting. CT is the second most commonly applied method for the detection of intracardiac thrombus. Our study shows that CT markedly improved left atrial thrombus assessment, especially left atrial appendage thrombus. This is supported by a previous study, in which CT showed outstanding performance with high sensitivity, specificity, positive predictive value, and negative predictive value in identifying left atrial appendage contrast filling defect (100, 81.0, 40.7, and 100%, respectively) compared to the gold standard (TEE and intracardiac echocardiography [ICE]) (17).

### Anticoagulation treatment

The overall anticoagulation prescription rate was 56.8% in our study, 45.1% were on warfarin therapy, and nearly half of the patients were off anticoagulation. Despite a relatively low prescription rate, an increasing trend in the anticoagulation rate is encouraging (Figure 5). Warfarin keeps its leading status as the most widely accepted anticoagulant owing to its relatively low price, definitive curative effect, and established adverse drug reaction. But warfarin therapy has non-negligible limitations, such as the interaction between foods and drugs, narrow therapeutic window, and demand for close monitoring. For HF patients with significantly decreased exercise tolerance, warfarin is at a competitive disadvantage as to patient compliance. Novel options are welcome to redress the balance, and a shift in inclination is partly reflected in the anticoagulant trend over time. Since 2012, the use of NOACs started to rise to become an increasingly popular option for clinicians, reflecting a gradually established and re-enforced awareness of these novel agents. Though pieces of evidence are accumulating regarding NOACs and nonvalvular AF anticoagulation, which indicate inferiority in stroke prevention (18, 19), there is still an evidence gap profiling safety and effectiveness of its role in intracardiac thrombus prevention and treatment in HF patients. Evidence regarding the effectiveness of NOAC application in this field is mostly based on case reports or small-scale studies, with anticoagulation duration varying from 30 days to 3 months (20–22). Hence, well-designed further studies are needed to explore the risk-benefit profile of NOACs in HF patients with intracardiac thrombus.

## Conclusion

As far as we know, this is the first study to focus on the clinical features of HF patients with intracardiac thrombus. It summarizes the underlying disease constitution, thrombus location, and status quo of anticoagulation in this population comprehensively; provides forceful evidence-based medical proof to better understand the clinical features of HF patients with intracardiac thrombus in China, and lays a foundation for continued intervention studies. In addition, it provides clinicians with significant clues to observe and intervene in this population.

This study has some limitations. It is a single-center observational study. Data were collected in a relatively high-level hospital, so the results are less representative of the whole population in China. Furthermore, its retrospective nature refrains us from obtaining all the details. It is of great necessity to carry out a registry study and thrombi-prevention study in this population to further determine whether patients will benefit from preventive anticoagulation therapy, especially in those with HF and sinus rhythm.

## Data availability statement

The raw data supporting the conclusions of this article will be made available by the authors, without undue reservation.

## Ethics statement

The studies involving human participants were reviewed and approved by Fuwai Hospital, National Center for Cardiovascular Diseases, Chinese Academy of Medical Sciences and Peking Union Medical College (CAMS & PUMC). Written informed consent to participate in this study was provided by the participants' legal guardian/next of kin.

## Author contributions

Conception and design of the study: MZ, LH, YZ, and JZ. Analysis and interpretation of data and drafting the article: MZ and LH. Acquisition of data: MZ, LH, LL, PT, LZ, XZ, BH, JF, YH, and QZ. Revising the manuscript critically for important intellectual content: YZ and JZ. All authors contributed to the article and approved the submitted version.

## Funding

This work was supported by the Key Projects in the National Science and Technology Pillar Program of the 13th Five-Year Plan Period (Grant Number 2017YFC1308300 to JZ), Beijing, People's Republic of China.

## Conflict of interest

The authors declare that the research was conducted in the absence of any commercial or financial relationships that could be construed as a potential conflict of interest.

## Publisher's note

All claims expressed in this article are solely those of the authors and do not necessarily represent those of their affiliated organizations, or those of the publisher, the editors and the reviewers. Any product that may be evaluated in this article, or claim that may be made by its manufacturer, is not guaranteed or endorsed by the publisher.
